# Perceptions of ‘Home Cooking’: A Qualitative Analysis from the United Kingdom and United States

**DOI:** 10.3390/nu12010198

**Published:** 2020-01-12

**Authors:** Susanna D.H. Mills, Julia A. Wolfson, Wendy L. Wrieden, Heather Brown, Martin White, Jean Adams

**Affiliations:** 1 Population Health Sciences Institute, Newcastle University, Newcastle upon Tyne NE1 7RU, UK; heather.brown@newcastle.ac.uk (H.B.); Martin.White@mrc-epid.cam.ac.uk (M.W.); 2 Department of Health Management and Policy, University of Michigan School of Public Health, 1415 Washington Heights, Ann Arbor, MI 48109, USA; jwolfson@umich.edu; 3 Human Nutrition Research Centre, Population Health Sciences Institute, Newcastle University, Newcastle upon Tyne NE1 7RU, UK; wendy.wrieden@newcastle.ac.uk; 4 Centre for Diet and Activity Research (CEDAR), MRC Epidemiology Unit, University of Cambridge School of Clinical Medicine, Box 285 Biomedical Campus, Cambridge CB2 0QQ, UK; jma79@medschl.cam.ac.uk

**Keywords:** cooking, home food preparation, diet, nutrition, health

## Abstract

Cooking at home is likely to be associated with benefits to diet and health. However, the nuanced perceptions and practices linked to different types of cooking are not yet fully understood. This research aimed to explore the specific concept of ‘home cooking’, using qualitative research from the UK and US. Data from two previously completed studies exploring cooking at home were combined and a new secondary analysis was undertaken using the Framework Method. Data in the first study were drawn from participants in the North East of the UK who were interviewed. Data in the second study were drawn from participants in Baltimore, US, who took part in focus groups. Data from a total of 71 adults (18 UK and 53 US), with diverse sociodemographic characteristics and experiences of cooking, were analysed. In both countries, participants distinguished ‘home cooking’ as a distinct subtype of cooking at home. ‘Home cooking’ was defined in terms of preparing a meal from scratch, cooking with love and care, and nostalgia. Cooking at home had a range of dimensions, and perceptions of ‘home cooking’ tended to focus on social and emotional associations. In future, public health initiatives might, therefore, highlight the potential social and emotional benefits of ‘home cooking’, rather than emphasising implications for physical health.

## 1. Introduction

The vast and growing international burden of ill health contributed by diet-related non-communicable diseases (NCDs) such as obesity, type II diabetes, and several types of cancer [1] has been paralleled by a decrease in time spent cooking at home in the majority of high-income countries [2,3]. Cooking at home may provide benefits for diet and health [4], and meals from out of home sources have been identified as a risk factor for higher energy and fat consumption, and lower micronutrient intake [5]. Nonetheless, to date, the evidence base has not proved definitive.

These observations have led some experts to conclude that promoting cooking at home, particularly traditional cooking ‘from scratch’ using basic ingredients, and encouraging the development of cooking skills, could offer one solution for addressing the concerning prevalence of diet-related NCDs [6]. However, currently, there is no consensus around the meanings and implications of cooking at home and, among the general public, interpretations span a wide range of ingredients, products and approaches to food preparation [7,8,9,10]. Lack of clarity over cooking terminology also persists [7,11,12]. The food environment of developed, industrialised countries such as the UK and US has evolved in recent decades, such that ubiquitous processed and convenience foods, and out-of-home food outlets, have made cooking a choice rather than necessity [13]. These transitions have also broadened potential interpretations of what it means ‘to cook’ [14]. Given the complexity of cooking, a shared understanding of terms around cooking at home is crucial for researchers, policy makers and practitioners in order that cooking behaviour and potential associations with diet and health outcomes may be accurately assessed, and the public may clearly understand public health messages regarding cooking. Similarly important is establishing realistic expectations concerning potential relationships between cooking at home, healthy eating and subsequent health outcomes.

Qualitative research has been used as an effective approach to develop insights into the multifaceted nature of home food preparation behaviours and perceptions [15,16,17,18,19,20,21,22,23,24,25]. In recent work, the phrase and concept of ‘home cooking’ has started to emerge as a novel, specific subtype within cooking food at home more generally [9], although characteristics have not been investigated further. There is currently a lack of clarity concerning what is meant by ‘home cooking’, and how perceptions may differ from the concept of cooking at home more generally. This is likely to be most effectively explored through in-depth qualitative analysis.

The work presented here aimed to explore the meanings and values embedded in ‘home cooking’, as distinct from other types of cooking at home, using data from two previous qualitative studies in the UK, and the US, that investigated cooking perceptions and behaviour. Combining data from these studies yielded a larger and more diverse participant sample and facilitated exploration of similarities and differences between two developed countries that have both undergone similar food system transitions, and face similar challenges regarding diet-related NCDs. The North East of England and Baltimore experience greater deprivation compared with respective national averages [26,27], and hence characterise conventionally under-represented population groups in research. In both the UK and US there have been calls to encourage cooking at home as a strategy to improve the public’s health [28,29].

The findings from the UK interviews [30] and US focus groups [9] have been published previously in terms of cooking perceptions, experiences and practices. This research presents new cross-country results from the combined subset of data that addressed perceptions and definitions of ‘home cooking’ specifically. Participants from both countries perceived ‘home cooking’ as a distinct subtype of cooking food at home more generally. Our results highlight the complexity of relationships between cooking and health, social and emotional values and indicate that ‘home cooking’ as widely perceived is more closely aligned with social and emotional benefits than physical health.

## 2. Materials and Methods 

This study is reported recognising the principles of the consolidated criteria for reporting qualitative research (COREQ criteria) [31]. The methods for the UK qualitative interviews [30], and US focus groups [9], have previously been published.

In brief, the UK study aimed to investigate home food preparation practices, experiences and perceptions through semi-structured interviews. Participants were purposively recruited from the city of Newcastle upon Tyne and surrounding area in the North East of England. A total of 18 participants were recruited through voluntary organisations, community, health and employment groups, social media and academic networks. A sampling matrix, using information on age, gender, ethnicity, marital status, household composition, and self-reported weight and interest and skills in cooking, was used to ensure a diverse sample. The matrix also included deprivation, using the 2015 Index of Multiple Deprivation (an area-based measure of deprivation using a range of routine statistics) [26]. Eligibility criteria for the study included being aged at least 16 years and acting as the main or shared main household food provider, as defined previously [32]. Interviews lasted between 35 and 80 minutes, and were directed using an iteratively developed topic guide, with questions related to views on cooking and cooking behaviours (see Supplementary File S1).

The US study aimed to explore perceptions of concepts related to cooking at home amongst American adults. Specifically, the research sought to examine individuals’ perceptions of what it means ‘to cook’, and to investigate important factors in how cooking is viewed and practiced. The study was undertaken in Baltimore, Maryland, where 53 participants were recruited from two urban neighbourhoods with contrasting sociodemographic populations and levels of neighbourhood socioeconomic disadvantage (based on median income and food access). In common with the UK study, this approach aimed to ensure a diverse participant sample. Volunteers responded to fliers posted at neighbourhood food outlets, libraries, churches and apartment buildings, and participated in one of seven focus groups. Inclusion criteria included being aged 18 years or over and living within the recruitment neighbourhood (based on self-report). Participants were accepted on a first come, first served basis. Group sessions lasted for approximately 90 minutes and were facilitated using an iteratively developed discussion guide, covering a wide range of topics related to perceptions of cooking and cooking behaviour (see Supplementary File S2).

In both the UK and US studies, basic field notes were taken, and audio recordings of the sessions were transcribed verbatim by a professional service. All subjects gave their informed consent for inclusion before they participated in the study and the study was conducted in accordance with the Declaration of Helsinki. The UK data collection was approved by Newcastle University Faculty of Medical Sciences Research Ethics Committee, application number 008585 2015. The US data collection was approved by Johns Hopkins University Institutional Review Board, application number 6027.

The UK interviews and US focus groups were undertaken discretely. Therefore, the lines of questioning used were tailored to the respective research methods and differed slightly. The secondary analysis presented here, combining datasets from the UK and US studies, provided a novel opportunity to explore in-depth the meanings and understandings of the specific concept of ‘home cooking’. Both primary data collection studies were similar in employing a qualitative approach and focusing on the perceptions and practices involved in preparing food at home. The combined analysis was enriched by comparing ‘home cooking’ between two distinct cultural settings, with differing national influences and sociodemographic characteristics.

Framework Analysis [33] was used to identify key themes concerning the meanings, values and importance of ‘home cooking’, with consideration for any influence of cultural context. Framework Analysis involves coding data with regard to the importance of emergent ideas and themes, in preference to the frequency with which they occur [34]. SM led the Framework Approach through an iterative process, assisted by JW. The main overarching emergent themes regarding ‘home cooking’ and associated key public health issues were identified using an evolving method. Interactions between focus group members were not explicitly analysed as part of this approach. In step one, the original full participant interview and focus group transcripts were re-read and reviewed. In step two, passages of text addressing ‘home cooking’ were extracted and merged, and an initial thematic framework was developed, guided by the research aims and incorporating themes identified previously through the interviews [30] and focus groups [9]. Key similarities and differences were identified between the UK and US participants’ home cooking perceptions and practices, and the emergent themes. Thematic codes were allocated iteratively in step three, and expanded and developed over time, following re-reading of transcripts and discussion between SM and JW. In step four, the tabulated themes were presented as a draft framework to the other coauthors, and then discussed and refined further on the basis of mutual consensus, according to associations, patterns and relationships identified within the data. The qualitative data analysis software NVivo 10 (QSR International Pty Ltd., Daresbury, Cheshire, UK) was used to facilitate the processes of data coding, management and analysis.

## 3. Results

The findings were drawn from a cross-country sample of 71 participants (18 UK and 53 US), with wide-ranging sociodemographic characteristics. One UK participant withdrew at an early stage in the study after expressing initial interest, leaving data from 71 participants for analysis (see Table 1). Due to the iterative nature of data collection in both UK and US settings, participant withdrawal did not prevent data saturation from being reached.

In both countries, the majority of participants were female. The US sample was more ethnically diverse than that drawn from the UK, and participants in the US were generally from older age groups than in the UK. A greater proportion of US participants described their marital status as single, compared with the UK. In terms of self-reported weight status, the majority of UK participants were overweight, whereas most US participants self-reported a normal or healthy weight.

Overall, participants in both the UK and US highlighted that practices and perceptions associated with cooking at home could vary greatly between individuals. Participants from both countries also perceived ‘home cooking’ as a distinct subtype of cooking food at home more generally. Emergent themes are described in further detail below, illustrated by participant quotations. The themes highlight the complexity of relationships between cooking and health, social and emotional values, and indicate that ‘home cooking’ as widely perceived is more closely associated with potential social and emotional benefits.

### 3.1. Cooking

Perceptions of cooking at home varied between individuals, in both the UK and US. Although a systematic analysis was not undertaken, these distinctions did not appear to be related to sociodemographic characteristics, and were largely consistent between countries, despite the social and cultural diversity within and between the two participant samples. In the US, however, greater emphasis was placed on the role of heat in the concept of cooking:

It’s just that for me, cooking does mean something was heated. US Participant 35, female.

Many participants recognised that cooking was a very personal activity, and others’ definitions could differ from their own. As one participant noted:

I think that cooking and home cooking means something different to everyone. US Participant 46, male.

For some participants, the term cooking at home encompassed all food preparation undertaken in the home, regardless of the extent of incorporating raw or basic ingredients. Viewed from this perspective, cooking at home included for example, heating a pre-prepared pizza in the oven, or adding hot water to a pot of instant noodles. Other participants considered that cooking should incorporate food skills and some degree of personal effort and involvement in the meal, such as chopping, combining and heating ingredients to produce a sauce.

Well actually, I’m more of a microwave man, if you want to call that cooking. US Participant 27, male.

### 3.2. Meanings and Values of ‘Home Cooking’

Throughout the cross-country sample, participants differentiated ‘home cooking’ as a distinct subtype of cooking at home, with unique properties. Three key overlapping themes emerged, which were consistent between participants from both the UK and US: cooking from scratch, demonstration of love and care, and nostalgia. These are presented in turn below, supported by participant quotations.

#### 3.2.1. Cooking ‘from Scratch’

Many participants described ‘home cooking’ as cooking ‘from scratch’ using basic ingredients that were raw or otherwise not processed. This definition of ‘home cooking’ excluded many convenience foods and ready-made products, though appeared to include widely used time-saving ingredients, such as dried pasta and tinned tomatoes. By focusing on the concept of cooking from scratch as the key principle of ‘home cooking’, emphasis was placed on the knowledge, general food skills and technical cooking skills required, and the creativity involved in producing composite meals from basic components. Many participants considered that other types of cooking at home also often included raw ingredients, but that these other types could incorporate convenience foods too. Thus ‘home cooking’ was a special subcategory, undertaken from scratch using basic ingredients only. 

To me home cooking means making something from scratch rather than from a pre-prepared item. For example I don’t think that if I buy a Pillsbury crust [frozen pastry] that I bring home and thaw and roll out, to me that’s not home cooking because I could have made the crust [pastry] from scratch. US Participant 46, male.

Make it from scratch and start on it from fresh fruit and vegetables or whatever you’re doing and you make it yourself. Rather than buying it and warming it up you make it yourself… UK Participant 12, female.

Preparing from scratch. So not convenience food, and not ready made chicken cooked from frozen, it’s like buying fresh stuff, cooking it from scratch. UK Participant 11, female.

#### 3.2.2. Demonstration of Love and Care

Participants also highlighted the meaning of ‘home cooking’ as a demonstration of love and care. In this context, producing a ‘home cooked’ meal was often an opportunity to fulfil a duty or desire to care for dependents or loved ones, and involved enacting the role of a provider. This description linked with the concept of preparing a meal from scratch, by identifying the personal effort inherently involved in ‘home cooking’, and hence the potential to demonstrate love and appreciation for others through the preparation of a ‘home cooked’ meal.

Home cooking? It’s almost one of those warming kind of expressions, isn’t it? You think of mum, you think of a farmer’s wife… It kind of engenders that kind of feeling about it… And, of course, you see that kind of thing outside pubs, don’t you? It kind of pulls you in, home cooked. It’s not saying it was made in a factory or processes. There’s love gone into it. So, to me that’s what that suggests… But yeah, yeah, it’s all that kind of warm, loving, tender, thoughtful preparation of food. UK Participant 3, male.

And also the other ingredient about home cooking is the love because it’s something about knowing that somebody took two hours to make this from scratch, winter soup with fresh garlic and you have to do the eggplant [aubergine] first and then you cook it with the other ingredients and it takes so long. One time I made a homemade soup for my pastor. And my pastor’s wife’s comment was, “Oh my goodness, Sister, you can taste the love in it”. US Participant 1, female.

You got to put your heart into it. Because when you [are] cooking a home cooked meal, you put love into it. You’ve seen people roll biscuits [baked goods, similar to scones] and how they shape them and how they want them to all look the same. They are like when you do a meatloaf you got to toss that thing and pat it. You got to put your heart into it. US Participant 40, male.

I know she put love and time and energy into that so I guess I still feel like that’s home made even if it has a different—it clearly has a different nutritional value. It clearly has a very different cooking style but I think it’s the love and the effort that went into it. US Participant 33, female.

#### 3.2.3. Nostalgia

A third theme that emerged from participants’ responses associated ‘home cooking’ with nostalgia. Here, participants’ previous personal experiences shaped their understanding of the nature of ‘home cooking’. Meals cooked at home in childhood or earlier in life were often recalled with fondness. The home setting—often perceived as a secure and loving environment—as the location of food preparation may also have contributed to positive sentiments associated with ‘home cooking’. Favourite meals or those frequently provided by a caregiver were often cited as typical examples of ‘home cooked’ meals. ‘Home cooking’ associated with nostalgia also often appeared to determine the type of cooking practices and meals that participants ideally desired to emulate themselves.

Yeah. I think it would probably be an image of what my mum would do. When I was growing up. And that was always a meal from scratch… We very rarely had anything like oven chips or pizza unless it was like a weekend treat. So it would normally be things like—it would always be like your typical kind of meat and two veg… UK Participant 2, female.

Like mince and dumplings and things. It makes me think of what your Nana [grandmother] would cook. UK Participant 10, female.

Now nobody whip up pancakes no more like they used to: put them on the frying pan, flip them up… Everything now comes quick. That’s not home cooked. It might be cooked at home but it’s not home cooked. US Participant 25, male.

Across all these three themes of: cooking from scratch, demonstration of love and care, and nostalgia, ‘home cooking’ was perceived as more time and energy intensive, and sometimes more difficult, than other types of cooking at home, and not necessarily always achievable on a daily basis. ‘Home cooking’ was also highly valued and seen as socially desirable. For participants from both the UK and US, ‘home cooking’ was closely tied to cultural identity and traditions, and relationships with family and friends.

It’s like a culture, like my own culture, home cooking my own food from back home where I come from… UK Participant 13, female.

That’s home cooking to me, doing it with your friends and family. UK Participant 14, female.

Whilst it was important for some participants that their definition of ‘home cooking’ included preparing meals from scratch or using basic ingredients, the strong emotional, social and cultural values of ‘home cooking’ were the defining qualities that differentiated ‘home cooking’ from other types of cooking at home.

### 3.3. Perceived Benefits of ‘Home Cooking’

The main potential benefits associated with ‘home cooking’ were generally based on social, cultural, and emotional gains, and occasionally with dietary advantages derived from using raw foods or traditional cooking techniques that were not reliant on highly processed ingredients. Participants’ accounts of ‘home cooking’ were often associated with positive memories, happiness and with overall wellbeing.

I love baking my cakes… More so if I’ve got… if I’ve just got my girls in, weekend… Because it involves them, you see. UK Participant 12, female.

Many participants described pleasure in cooking a meal for others, and a sense of satisfaction in creating a dish from basic ingredients. They felt that ‘home cooking’ was important for fostering strong and loving personal connections. Participants stated:

So in the house, as long as I’ve cooked the main meal, you feel comfortable because you can have anybody sit and have a meal with you. UK Participant 7, female.

I love home cooked food. I came from a family that [were] from the south, North Carolina, South Carolina, Virginia. I ate the gamut of foods, rice, potatoes, whatever. And I like for my food to taste like it was cooked with just a little bit of love going in that gravy, going over them potatoes. It has a certain taste. US Participant 9, female.

There were few perceptions of ‘home cooking’ directly related to more clinical concepts of dietary quality and health, such as this participant’s view:

To me, what defines home cooking is that you put all of the ingredients in it and the main thing is it’s not pre-packaged, pre-prepared or preserved because the stuff that they’re adding to food now to increase the shelf life, the high fructose corn syrup… and like she said, even what makes the frozen foods last so long is they add a lot of sodium to it. You don’t have that extra stuff going into your diet. You know what you have in it. It’s fresh… And I think that helps even your health because everything affects your health, your mood, the way you feel. I think home cooking is an important necessary ingredient for healthy living. US Participant 1, female.

In both the UK and US, perceptions of ‘home cooking’ usually had little association with eating healthily and promoting physical health. Although cooking at home more generally may be used as a strategy to control one’s diet and attempt to eat more healthfully [35], participants in this study did not make a similar connection with the specific concept of ‘home cooking’. Rather, participants tended to define ‘home cooking’ in terms of the involvement of basic ingredients, level of personal effort, and reminiscence of meals with emotional and cultural significance in times gone by. 

## 4. Discussion

Qualitative data from combined UK interviews and US focus groups investigating cooking at home showed that in both countries, ‘home cooking’ was identified as a distinct subtype within the category of cooking at home more generally and values ascribed specifically to ‘home cooking’ were similar. Three key themes concerning the meanings and values inherent in ‘home cooking’ were identified, namely cooking from scratch, demonstration of love and care, and nostalgia.

These insights provide additional opportunities for exploring potential relationships between cooking, diet and health. Cooking at home and developing cooking skills have been promoted in both the UK and the US as strategies to help improve diet quality and the prevalence of dietary related NCDs [6,28,29]. However, in this research, participants did not specifically link diet quality or physical health with the concept of ‘home cooking’, and instead focused more on social and emotional dimensions, such as nostalgia.

### 4.1. Strengths and Limitations

This study benefits from a number of strengths. The study design is novel, using qualitative data from two countries to focus specifically on ‘home cooking’ as a subcategory of cooking at home. The UK and US have experienced similar changes in the wider food environment in recent decades and face comparable challenges regarding the high and escalating prevalence of dietary related NCDs. Therefore, exploring the meanings and perceptions of ‘home cooking’ among participants from these two countries provides an opportunity to inform public health promotional messaging, and development of potential future cooking interventions, in both localities. The few previous international comparative studies of cooking have largely been quantitative, and addressed for example time spent on cooking [36], attitudes to food and the role of food [37], domestic cooking habits in general [17], and overall meal patterns and cooking practices [38]. The UK and US data analysed here were collected using topic guides with open-ended questions, which were updated and revised as the research progressed, and through further probing of emerging ideas and concepts. The process is, therefore, likely to have been comprehensive, enabling data saturation to be reached, and producing in-depth data on cooking at home. The Framework Approach [33] was used to systematically compare and contrast participants’ perspectives, and to iteratively analyse the overarching themes from the merged UK and US datasets, thereby enabling detailed exploration of key findings. 

However, the research is also subject to limitations. The combined qualitative data sample was drawn from two separate populations, using different data collection methods. Interviews and focus groups tend to have distinct aims with regards to participant interaction and reaching consensus [39], which could have affected the subsequent integration of data from these two sources. The primary UK interviews and US focus groups did not specifically seek to address perceptions of ‘home cooking’, therefore it is possible that further targeted probing could have provided additional insights regarding this concept. UK participants were all required to be a shared or main household food provider, whereas this inclusion criterion was not specified for US participants, which could have influenced the nature of the data collected. The range of opinions expressed regarding ‘home cooking’ may also potentially have been partly attributable to the diversity of participants involved. 

Data analysis in this combined study required data merging, which created an extra analysis step, with associated additional potential for introduction of researcher bias. Nonetheless, at all stages of the research we endeavoured to ensure reflexivity and confront any prior biases and assumptions brought to the study. This was undertaken by cross-checking the interpretation of emergent themes between coauthors and considering the research findings in the context of existing related literature.

In common with the majority of qualitative research studies, the findings presented here were generated from a relatively small number of participants and may not necessarily be more widely generalisable. In particular, the nature of cooking at home, and meanings and values attached to associated terminology, are likely to differ from those in less developed countries with more traditional cooking cultures. However, the concordance in themes between participants in both the UK and US, and support for findings from previous studies, suggest that the key concepts identified here are likely to be transferable to other similar population groups, especially those of Northern European heritage.

### 4.2. Interpretation and Implications

Overall evidence to date indicates that cooking at home more generally may be associated with diet, health and social benefits [4]. However, cooking behaviours are complex and varied [7,40,41], exhibiting a spectrum from heating up pre-prepared food in a microwave, to producing elaborate meals from raw ingredients [9]. These practices are likely to span a range of healthfulness, determined by factors including the ingredients involved and food preparation methods used. 

This research indicated that health did not appear to be a key consideration within the concept of ‘home cooking’. However, potential social advantages were noted. Participants described demonstrating love and care for others through ‘home cooking’ and reflected on pleasurable ‘home cooked’ meals shared with family and friends in the past. This supports evidence that cooking may help facilitate bonding between families and friends [30]. The familiar home environment is likely to provide positive connotations for undertaking food preparation and eating meals together. ‘Home cooking’ could, therefore, offer benefits through non-dietary routes such as mental health and wellbeing, consistent with prior work underscoring the potential social and emotional merits of sharing a meal with others, particularly in the family setting [42,43,44]. 

Despite the caveats identified around potential diet and health implications of ‘home cooking’ specifically, the broader concept of cooking at home remains integral to most people’s dietary patterns. Cooking from basic ingredients has to a certain extent been declining in high-income countries in recent years [2,3], but nonetheless surveys indicate that the majority of the population in the UK [45] and US [46] still regularly eat meals cooked at home. Putative gains for diet and health have been identified from cooking at home more generally [4]. Cooking, therefore, cannot be disregarded in relation to public health, and contemporary campaigns frequently refer to the specific concept of ‘home cooking’ [47].

Overall, the findings presented here suggest that public health initiatives to tackle diet-related NCDs may involve the promotion of cooking at home as a strategy offering potential diet, health and social benefits. However, such messages should advocate clearly and carefully, and might most successfully focus on potential social and emotional gains. Public health nutrition campaigns should aim to integrate the promotion of cooking with broader dietary guidance, such as recommendations for the consumption of wholegrains and fruit and vegetables [47]. Training is also likely to be required to develop technical cooking skills, broader general food skills, and food literacy, providing ‘the tools needed for a healthy lifelong relationship with food’ [48] (p. 54). This may be effectively achieved by expanding home economics education and improving links with public health, in order to grow capacity for healthy cooking at home.

### 4.3. Wider Research Context

To our knowledge, this is the first study to directly elicit and describe differences between the specific meanings and values inherent in ‘home cooking’, and those associated with cooking at home more generally. Previous research has indicated that cooking practices and perceptions are shaped both by individual determinants, such as socioeconomic status [49,50], and by cultural factors [20,46]. This research used evidence from two countries to identify strong similarities between concepts of ‘home cooking’ in different cultural contexts, and between participants with varied sociodemographic backgrounds. Clarifying whether these concepts differ systematically according to different participant characteristics and cultural background was beyond the scope of this qualitative study and offers an important area for further exploration.

This study builds upon previous evidence indicating that cooking food at home is not one single process or entity, but rather spans several continua of perceptions and behaviours, with associated implications for health [4,9,12]. This concept is not likely to be explored satisfactorily through quantitative ‘tick-box’ style data collection, unless future questions are designed to capture the nuance and complexity of different types of cooking. Lack of clarity in research studies, without adequate explanation of key terms and/or capacity to highlight varied perceptions and practices, is liable to lead to varied and potentially invalid findings. If ‘home cooking’ confers fewer positive implications for physical health than cooking at home more generally, failure to discriminate between the two may lead to dilution of potential benefits from cooking at home more broadly.

The findings reported in this study support previous research indicating that ‘home cooking’ is perceived as a ‘proper’ or superior method of cooking [51]. However, our study did not specifically seek to investigate whether any other subtypes of cooking exist within cooking at home more generally. Further insights into the presence or absence of such subtypes, and their associated terminology, characteristics, and implications for health, would be likely to prove valuable.

## 5. Conclusions

This cross-country study combined qualitative data from the UK and US, highlighting ‘home cooking’ as a concept distinct from cooking at home more generally. Three key emergent themes regarding the meanings and values associated with ‘home cooking’ were identified: cooking ‘from scratch’ using basic ingredients, demonstration of love and care, and nostalgia. These themes suggest that dietary health concepts are not necessarily embedded within interpretations of ‘home cooking’, and greater emphasis is placed on social and emotional associations. Given that cooking at home more generally plays a fundamental role in societal eating patterns, and may offer diet, health and social benefits, public health initiatives should continue to promote cooking at home. However, further research exploring definitions, perceptions and experiences associated with different potential subtypes of cooking at home is required.

## Figures and Tables

**Table 1:** Characteristics of interview and focus group participants involved in the study.

Participant Characteristics	United Kingdom (UK) n (%)	United States (US) n (%)
**Total number**	18	53
**Age (years)**		
**<30**	3 (17)	8 (15)
**31–45**	11 (61)	7 (13)
**46–55**	0 (0)	16 (30)
**56–65**	1 (5)	14 (27)
**≥66**	3 (17)	8 (15)
**Gender**		
**Male**	5 (28)	14 (26)
**Female**	13 (72)	39 (74)
**Race/Ethnicity ^i^**		
**White**	15 (83)	16 (30)
**Black**	1 (6)	35 (66)
**Asian**	0 (0)	2 (4)
**Pakistani or Bangladeshi**	2 (11)	Not asked
**Hispanic**	0 (0)	0 (0)
**Marital Status**		
**Single**	4 (22)	26 (49)
**Married**	5 (28)	8 (15)
**Living with partner**	6 (33)	10 (19)
**Divorced/separated/widowed**	3 (17)	9 (17)
**Weight status ^ii^**		
**Underweight**	0 (0)	4 (8)
**Normal/healthy weight**	8 (44)	32 (60)
**Overweight**	10 (56)	9 (17)
**Overweight by >20 pounds**	Not asked	7 (13)

^i^ Self-reported. In the UK sample, participants described their race/ethnicity via an open-ended question. In the US sample, response categories were White, Black, Hispanic, or Asian. ^ii^ Self-reported. In the UK sample, response options were underweight, normal, or overweight. In the US sample, response options were underweight, healthy weight, overweight, or overweight by >20 pounds. One US participant declined to respond.

## Supplementary Materials

The following are available online at https://www.mdpi.com/2072-6643/12/1/198/s1, File S1: Interview topic guide, File S2: Focus group topic guide.
