# Supplementary material for: Perceptions of ‘Home Cooking’: A Qualitative Analysis from the United Kingdom and United States
Source: Nutrients. 2020 Jan 12;12(1):198. doi: 10.3390/nu12010198 (PMC7019500; doi:10.3390/nu12010198)
Supplement: Supplementary file 1 [file nutrients-12-00198-s001.zip › Supplementary file 1.pdf]

Interview topic guide

The aim of these interviews is to understand how adults prepare food at home, what factors influence their behaviour, and the barriers to home cooking. The research also aims to identify facilitators and barriers for cooking healthily. The interview topic guide will be developed on the basis of findings from a systematic review of determinants and outcomes of home cooking, undertaken in phase one of this programme of research. The topic guide will also be iteratively revised according to learning and feedback gained from piloting and early interviews. It is anticipated that each interview will last for up to one hour. The questions will be semi-structured, and the examples below provide an indication of the proposed areas for questioning.

Introduction

Interviewer introduces themselves, and the aims of the study.

Ground rules:

- Participant is free to state at any time if they feel uncomfortable with questions or want to stop the interview.
- The interview will be audio recorded and the interviewer will make brief notes. Both will be anonymised after the interview.
- There are no right or wrong answers and all responses are valid.

Prompts

- Tell me about the meals that you’ve had over the past week. Did you cook any meals? What did you prepare?
- Tell me about your cooking photos.

General questions

- Tell me about your eating habits and your usual home food preparation behaviour.
- What does home cooking mean to you?
- How do you feel about cooking (eg enjoyable, a nuisance)
- Are there particular aspects that you do or don’t like? Why?
- How would you describe yourself as a cook? (eg good, bad, OK, boring, safe, adventurous, nervous, healthy, unhealthy)
- Are you a confident cook? Why/why not?

Household

- Who prepares meals in your household?
- How often does the household have a cooked meal?
- What sort of meals do you have? (favourites/last night)

- During a typical week, how often do you cook a meal from scratch? (for example using raw chicken, spices and vegetables to make a chicken curry)
- During a typical week, how often do you cook a meal using pre-prepared ingredients? (for example using dried pasta and ready-made sauce)
- During a typical week, how often do you have ready meals and/or takeaways?
- During a typical week, how often do you skip meals or have snacks instead?
- What factors influence these choices?
- Which main dish do you consume most often? (to inform phase 3 analysis)
- Where do you eat your meals? Why?
- Do you eat with anyone else? Who and why?

Determinants

- What influences your eating habits?
- What factors make it easier for you to cook? (eg lots of time, good kitchen facilities, enjoyment)
- What factors make it difficult for you to cook? (eg too busy, ingredients are expensive, don't have necessary skills)
- What factors would you like to change?
- Who has an impact on what and how you cook?
- Has your home food preparation behaviour changed over time? Why/why not? Would you like to change in the future?

Health

- What does healthy cooking and eating mean to you?
- Does the healthiness of foods influence your cooking and eating patterns?
- What comes before health in your priorities and why?

Skills, facilities and shopping

- What cooking and storage facilities do you have? (eg hob, fridge, cupboards)
- What utensils and equipment do you have? (eg pans, knives, chopping board)
- Do you have any cooking skills? If so, which? (show card of different skills eg frying, grilling, roasting)
- Where did you learn? (eg mother, school, picked it up as you went along)
- Do you use recipes? Where from and why? Do you find them easy to follow?
- Do you plan your meals and/or food shopping in advance?
- Who does the food shopping and why? How often do you go shopping? What kind of shops/online? How do you decide what to buy?

Wrap up

Is there anything else you'd like to mention that we haven't covered?

Close; thank the participant; and provide debriefing sheet and 'thank you' voucher.
