# Supplementary material for: Perceptions of ‘Home Cooking’: A Qualitative Analysis from the United Kingdom and United States
Source: Nutrients. 2020 Jan 12;12(1):198. doi: 10.3390/nu12010198 (PMC7019500; doi:10.3390/nu12010198)
Supplement: Supplementary file 1 [file nutrients-12-00198-s001.zip › Supplementary file 2.pdf]

Approved: October 9, 2014 **IRB No.**: 6027

Appendix D
Focus Group Discussion Guide*

I. Introductions

Introduce ourselves (moderator and the note taker): We are graduate students at Johns Hopkins in the School of Public Health. We are conducting a research study about how people think about how people prepare food and how they think about cooking. The goal of this discussion group is to learn more about food preparation habits, what factors influence how you prepare food and how you think about cooking. There are no right or wrong answers.

Ask participants to introduce themselves and provide a first name or pseudonym.

Icebreaker: What is your favorite food or cooking memory?

II. Food preparation practices and history

1. What was the last meal you cooked? Why did you choose it? How did you make it?

    Probe: Is this typical for you?

    Probe: Do you think this is typical of most people?

    Probe: What influences the way you prepare meals? Bring up food access, food prices, taste preferences, time, enjoyment, ease of preparation?

2. Is the way you cook similar to the way your family cooked when you were growing up?

    Probe: If yes, in what way is it different?

    Probe: If yes, why is it different?

    Probe: What about your family today?

3. What kinds equipment and food preparation techniques do you use?
    (use photos of different equipment, products and techniques to illustrate)

*Questions are examples of the kind of questions that may be asked. Later focus groups will solicit feedback on ideas shared in the earlier groups.

4. How did you learn how to cook?

   Probe: Ask about mothers, grandmothers, fathers, school, home economics classes, recipes/cookbooks, cooking shows?

5. How have your cooking habits changed over time?

   Probe: Have there been specific things in your life that have changed the way you prepare food?

III. Perceptions of cooking

6. How do you feel about cooking?

   Probe: Do you enjoy cooking? Does it stress you out? Is cooking work? Is it fun?

   Probe: How do you feel about the way you cook? Do you think you are good cook? Are you confident in your cooking skills?

   Probe: What are your favorite and least favorite things about cooking?

7. What does home cooking mean to you?

   Probe: How is home cooking different from cooking in general? If at all?

   Probe: What about scratch cooking?

   Probe: In surveys, people report that they cook very frequently, like six or seven nights a week. Based on your experience what kinds of food preparation activities do you think they are talking about?

8. What kinds of things do you consider when deciding whether or not something was homemade?

   Probe: What about packaged foods or frozen foods?

9. How would you describe meals made with these things? Are they homemade? Is this cooking? Or something else? Is there no difference?
   (use photos or actual products as props to illustrate and guide the discussion)

*Questions are examples of the kind of questions that may be asked. Later focus groups will solicit feedback on ideas shared in the earlier groups.

10. What are some of the challenges you face when it comes to cooking?

    Probes: Ask about confidence, time, skills, enjoyment, cost, food access, meal planning/organization, preferences of family members.

    Probe: What equipment is necessary to cook?

11. Is cooking important to you? Why or why not?

    Probe: Is it important for health? For family? For some other reason?

12. Some people promote eating more home cooked meals as a way to eat healthier. What do you think about this recommendation?

    Probe: What is the most useful way to help people cook more at home?

    Probe: For you personally, or others in your community, what changes would you like to see that would help people cook more?

IV. Conclusion

We've covered a lot and I appreciate your insights. I want to make that everyone has an opportunity to provide any additional ideas.

13. Is there anything else you would like to add or that you think we should know about cooking

*Questions are examples of the kind of questions that may be asked. Later focus groups will solicit feedback on ideas shared in the earlier groups.
